# Irrigation and passive drainage of pancreatic stump after distal pancreatectomy in high-risk patients: an innovative approach to reduce pancreatic fistula

**DOI:** 10.1007/s00423-020-02012-9

**Published:** 2020-10-21

**Authors:** Olga Adamenko, Carlo Ferrari, Jan Schmidt

**Affiliations:** 1 Hirslanden Hospitals, Kappelistrasse 7, 8002 Zürich, Switzerland; 2 Università degli Studi di Milano, Via Festa del Perdono 7, Milan, 20122 Italy

**Keywords:** Irrigation, Drainage, Distal pancreatectomy, Fistula, Complications, POPF

## Abstract

**Introduction:**

Postoperative pancreatic fistula (POPF) represents the most common form of morbidity after distal pancreatectomy (DP). The aim of this study was to illustrate an innovative technique of irrigation and passive drainage to reduce clinically relevant POPF (CR-POPF) incidence in high-risk patients undergoing DP.

**Material and methods:**

Twelve consecutive high-risk patients received irrigation and passive drainage of the pancreatic stump with a Salem sump drainage after DP. The Salem sump was irrigated with 100 ml/h of Ringer solution for 2 postoperative days (POD). In the case of low-drain amylase and lipase levels on POD 3, the irrigation was reduced to 50 ml/h. Persistence of low-drain pancreatic enzymes on POD 4 allowed for interruption of irrigation and subsequent removal of drainage from POD 7 onward in the absence of evidence of any pancreatic fistula.

**Results:**

Overall, 16.6% of the patients experienced a grade 3 or higher surgical complication. We experienced only one case of POPF: the fistula was classified as grade B and it was managed with radiologic drainage of the fluid collection. We did not experience any case of re-operation nor in-hospital mortality.

**Conclusions:**

Irrigation with passive drainage of the pancreatic stump after DP is an interesting approach for CR-POPF prevention in high-risk patients.

## Introduction

Distal pancreatectomy (DP), with or without splenectomy, is the procedure of choice for benign, malignant, inflammatory and traumatic pathologies affecting distal pancreas. Data retrieved from the most recent literature report a mortality rate lower than 5% in tertiary centers [1–4]; however, the burden of postoperative morbidity remains still high, ranging from 22 to 64% of cases [1, 2, 4–6]. Among the postoperative complications, the most frequent event is represented by postoperative pancreatic fistula (POPF), with an incidence ranging between 3 and 40% [3, 5, 7–9]. In contrast with established risk factors for prediction of POPF development after pancreaticoduodenectomy, few validated (and widely accepted) algorithms exist for bed-side, clinical risk stratification for POPF development following DP. Being unable to stratify patients according to their risk factors, the literature rarely reports postoperative outcomes for the different subpopulations at high, intermediate or low risk of POPF development [4, 5, 10–14]. Moreover, widely accepted strategies to reduce the incidence of POPF are lacking as well. Among these, intraoperative drainage placement and their management have been thoroughly questioned, but often leading to conflicting results [15]. In this paper we promote an innovative technique of irrigation and passive drainage in patients at higher risk of developing POPF after DP.

## Materials and methods

### High-risk pancreas

Due to the lack of widely exploited models for the recognition of subjects more prone to develop POPF after DP, we based the inclusion of our patient on the most updated data from the literature combined with our clinical experience. Therefore, we included in the high-risk category all those patients presenting both the following compulsory characteristics:

- Thick pancreas at the site of transection. According to our experience, a thick pancreas at the site of transection is the most prominent risk factor for the development of POPF. This belief is supported by several findings coming from the literature [3, 8, 16–18]. Two studies from Sugimoto et al. [8, 19] highlighted the relationship between pancreatic thickness and development of POPF after DP. Same results could be found in the work of Fukuda et al. [17]; moreover, the Japanese group demonstrated that POPF rates increased significantly with increasing pancreatic thickness. According to these findings, we included in this category all patients having a preoperatively measured pancreas thickness on CT scan at the approximate site of transection greater than 12 mm.
- Soft pancreatic texture. Similarly to pancreaticoduodenectomy, soft pancreatic texture has been recognized as a risk factor for development of POPF also in DP [3, 20–22]. The results from Xia et al. [21] demonstrated the association between soft pancreatic parenchyma with both overall POPF and clinically relevant POPF (CR-POPF). The association between pancreatic texture and CR-POPF remained statistically significant also at the multivariate level. In the systematic review and meta-analysis by Peng et al. [20], the pooled odds ratio and 95% confidence interval of 14 articles supported the association between soft pancreatic texture and POPF. The work by Mendoza et al. [3] failed to identify soft pancreatic parenchyma as a risk factor by itself; however, their results showed an increased incidence of POPF development when a soft pancreas was associated with a thick stump.

As additional, facultative criteria for the recognition of high-risk patients, we included:

- High BMI. Several studies report an association between obesity and increased incidence of POPF after DP [1, 5, 7, 11, 20–24]. The study of Vanbrugghe et al. [5] identified an association between CR-POPF and both BMI ≥ 25 kg/m^2^ and a visceral fat area ≥ 92 cm^2^ measured on preoperative CT scan. Moreover, the latter was found to be an independent predictor of CR-POPF also at the multivariate analysis. In the paper of Yamashita et al. [25], BMI ≥ 25 kg/m^2^ was independently associated with the development of severe postoperative complications. A strong contribution was given, again, by the meta-analysis from Peng et al. [20], which highlighted how patients with elevated BMI are more prone to suffer from POPF.
- Multiorgan resection. Multivisceral resections increase the length and the complexity of the surgical procedure, therefore augmenting the risk of postoperative complications. Data from the literature confirmed the association of multiorgan resection with both POPF [1, 16, 26] and increase in morbidity rates [27, 28] after DP. In particular, the association with intestinal resection increases the risk of developing a severe postoperative complication such as pancreatico-intestinal fistula.

Whenever a patient met these criteria, the surgical procedure and, most importantly, the drainage allocation was performed as follows.

### Surgical procedure

In case of tumors not extending beyond the pancreatic capsule, the laparoscopic approach was the procedure of choice and it was performed in a standardized fashion.

After arranging the trocars in a semi-circular fashion in the superior abdomen, with the optic trocar positioned in the supraumbilical region, the gastrocolic ligament is opened with an ultrasound device (Thunderbeat™; Olympus, Japan) caudally to the marginal arcade of the great gastric curvature. After gaining access to the lesser sac, the transverse colon is mobilized and displaced caudally: at this point, the hepatic retractor is placed under the posterior gastric wall, which is then lifted cranially to better expose the pancreatic surface. An intraoperative ultrasound is performed in order to localize the pancreatic lesion. The inferior pancreatic margin is dissected with Thunderbeat™ until the superior mesenteric vein is exposed. The splenic artery is then isolated and sealed with a medium-size Hem-o-lok™ clip (Weck Surgical Instruments, Teleflex Medical, Durham, NC, USA) about 1 cm medially with respect to the pancreatic parenchyma transection plane. The splenic vein is exposed and isolated. The pancreatic parenchyma is underpassed and sectioned by using an EndoGia™ stapler (Covidien, Boulder, CO, USA) with black, reinforced cartridge. Subsequently, the splenic vein is transected with EndoGia™ stapler loaded with a vascular cartridge. The body-tail of the pancreas is lateralized toward the left side and dissected along the plane laying ventrally to the Gerota’s fascia. Lymphadenectomy is carried out in the peripancreatic area and along the common hepatic artery, celiac trunk and splenic artery. The short gastric vessels and the spleno-colic ligament are sectioned with Thunderbeat™. The spleen is freed from its retroperitoneal adherences, until reaching the dissection plane previously prepared, ventrally to the Gerota’s fascia. The en-block surgical specimen including pancreas, spleen, and lymphnodes is positioned into an endobag, and it is retrieved via a Pfannenstiel incision.

When the tumor extended beyond the posterior pancreatic capsule on preoperative CT scan, the preferred approach was an open posterior radical antegrade modular pacreatosplenectomy (RAMPS) [29]. Right after the opening of the omental bursa, division of the short gastric vessels, and lateralization of the stomach, the posterior RAMPS establishes the underpassage and division of the pancreatic gland at the head-to-body junction. After the exposure of the celiac trunk and the superior mesenteric artery, lymphadenectomy is carried out along the common hepatic artery and celiac trunk and along the origin of the splenic artery. After dividing the splenic vessels, pancreatic resection is carried out from right to left, therefore removing into an en-block specimen the pancreatic body and tail, the spleen, the left adrenal gland, and all tissues included within the left margin of the aorta, the diaphragm, and the postero-lateral abdominal wall.

In the case of tumor extending beyond the anterior pancreatic capsule or infiltrating surrounding organs, such as stomach, colon, or mesocolon, an open, left-to-right approach was performed as follows. The abdomen is entered via a median, superior laparotomy. The coloepiploic detachment is performed and access to the lesser sac is obtained. The left colic flexure is then mobilized until the plane laying anterior to the Gerota’s fascia; the short gastric vessels are dissected with an ultrasound device (LigaSure Impact™, Covidien, Boulder, CO, USA). The body and tail of pancreas are then mobilized until the exposure of superior mesenteric vessels is achieved. The splenic vein is transected with an EndoGia™ stapler with vascular cartridge; the splenic artery is sealed with a medium-size Hem-o-lok™ clip about 1 cm medially with respect to the pancreatic parenchyma transection plane. Lymphadenectomy is carried out in the peripancreatic area and along the common hepatic artery, celiac trunk, and splenic artery. In case of suspected or confirmed malignancy, the pancreatic tail and body are dissected leaving a macroscopic 3 cm margin from the tumor: the parenchyma is then resected by using an EndoGia™ stapler with black, reinforced cartridge. The surgical specimen containing body-tail of pancreas, spleen, and lymphnodes is removed en-block.

Despite the procedure performed, some common steps are carried out independently from the surgical approach:

- The falciform ligament is mobilized and sutured with 3-0 Prolene (Ethicon, Somerville, NJ, USA) on the pancreatic stump.
- The greater omentum is partially mobilized and is positioned in the splenic lodge.
- Intraoperative frozen section is carried out to confirm the absence of microscopic tumor on the resection margins. In case positivity of resection margins for tumoral cells is confirmed, the resection of the pancreatic parenchyma is extended and a new intraoperative frozen section is carried out.
- A 15-Ch Salem sump irrigation tube with passive drainage (Covidien, Boulder, CO, USA) is positioned near the pancreatic stump and it is fixed, firstly to the peritoneum with 4-0 Vicryl Rapide and then to the skin with a 0-0 Ethibond (Ethicon, Somerville, NJ, USA) purse string suture.
- A drainage catheter (Cystofix; Braun, Melsungen, Germany) is positioned in the lower pelvis via a separate access in the left flank.

**Fig. 1:**
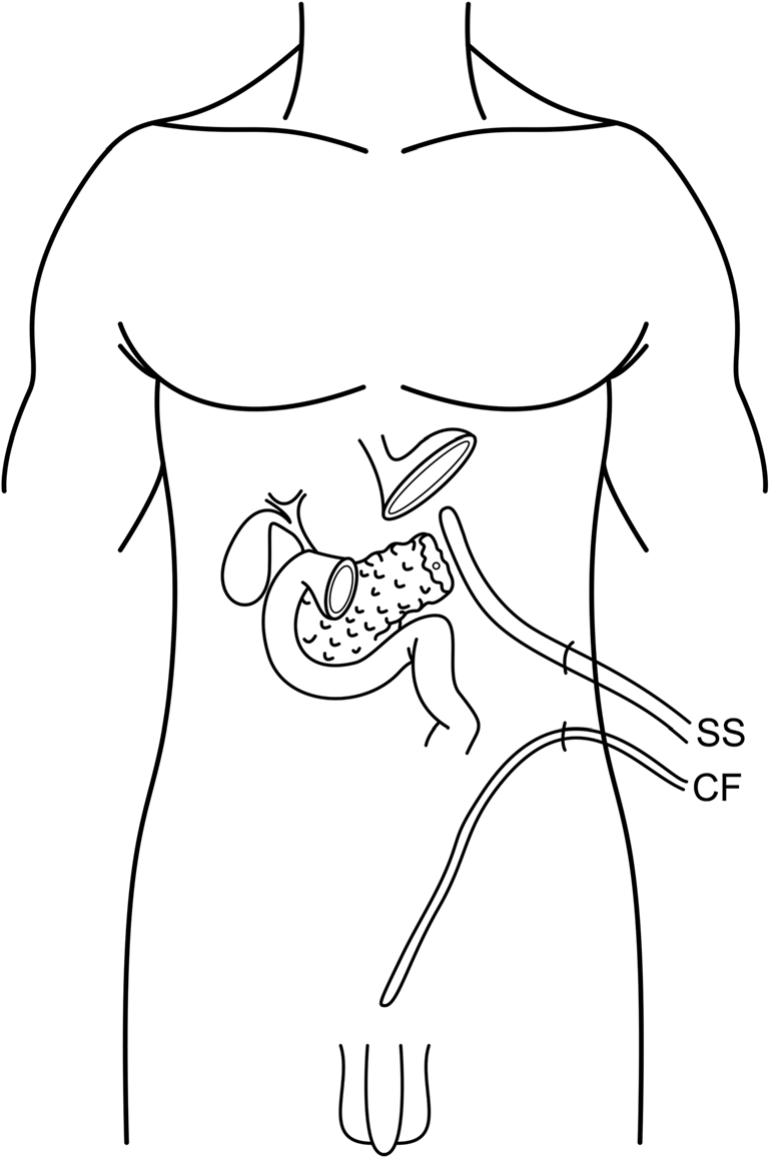
Schematic representation of the drainage positioning (SS, Salem sump drainage; CF, Cystofix drainage)

A schematic representation of drainage positioning is shown in Fig. 1.

Every patient received 0.6 mg of pasireotide (Signifor®, Novartis) the evening before and on the morning of the surgical procedure. Patients then received 0.6 mg pasirotide three times a day, until postoperative day (POD) 5.

### Postoperative drain management

The Salem sump was flushed with 100 ml/h of Ringer solution for 2 postoperative days; the drainage of the collected fluid and possible pancreatic juices occurred by passive gravity. The quality of the drained fluids was inspected every day in order to recognize sinister appearance. Drain amylase and lipase values on POD 3 lower than three times the serum level in that given patient on the same day, allowed for reduction of the flushing speed with Ringer solution to 50 ml/h. In case the levels of amylase and lipase remained under the aforementioned cut-offs also on POD 4, Ringer irrigation was interrupted and only passive drainage was allowed. New biochemical tests were carried out on drained fluids on POD 5 and 6: Salem sump drainage and pelvic drainage catheter were routinely removed from POD 7 onward in the absence of clinical or biochemical evidence of any pancreatic fistula, within a timeframe compatible with adequate loss of strength of the absorbable suture fixing the tip of the drainage. In order to obtain the real concentrations of pancreatic enzymes in the drained fluids, irrigation was interrupted every day for 2 h, and bags collecting the drainage fluids were changed before retrieving the samples needed for biochemical analysis.

### Statistical analysis

Association between POPF or severe complications and categorical variables was investigated using *χ*^2^ test, with a significance level of 0.05. When parametric assumptions were met, instead, Student *t* two-tailed test was used to compare means of continuous variables; otherwise, Mann-Whitney *U* test was performed. Data are mean ± standard deviation, unless otherwise stated. A significance level of 0.05 was chosen. Given the paucity of subjects in our sample combined with the fact that a regression analysis generally needs a number of outcomes of interest at least ten times higher than the number of the variables that can be studied [30], we decided not to perform any multivariate analysis for our study population.

## Results

### Data collection

A total of 21 patients underwent DP between September 2015 and July 2019 at Hirslanden Klinik or Klinik Im Park (Zürich, Switzerland).

All patients were operated by a single surgeon (J. S.) experienced in pancreatic resections (> 945 pancreatic resections), thus removing any potential bias for performing this study in a low-volume center for pancreatic resections.

Among the 21 patients, we included in the analysis 12 consecutive subjects at high risk to develop POPF. For each patient, a standardized procedure was carried out with allocation of an irrigation drainage as explained in the previous section.

Postoperative complications were graded according to the Clavien-Dindo (CD) classification [31]: grade 3 or higher complications were recorded as “severe.” All POPFs were categorized according to the classification proposed by the International Study Group of Pancreatic Fistula (ISGPF) [32].

We recorded and included in this analysis all perioperative variables potentially associated with the development of POPF or severe complications according to the literature and to our experience. Data were retrieved and analyzed from a prospectively maintained database (Excel, Microsoft Corp, Redmond, WA). Statistical analysis was performed using SPSS ver.24 for Macintosh (IBM, Chicago, IL).

### Descriptive characteristics

Demographic, clinical, and perioperative data are summarized in Table 1. The indication for surgical resection was adenocarcinoma, neuroendocrine tumor, or IPMN of the pancreatic body or tail in 4 (33.3%), 3 (25%), and 3 (25%) cases, respectively. In one case, distal pancreatectomy was performed in order to resolve a persistent pancreatic fistula after the enucleation of an insulinoma of the pancreatic tail. One patient underwent distal pancreatectomy because of the localization of renal cell carcinoma metastases in the pancreatic body-tail junction. Concomitant splenectomy was performed in 12 (100%) cases; multivisceral resection occurred in 5 (41.6%) cases because of multiple organ infiltration. Vascular resection or intervention was performed in 3 (25%) cases: one patient underwent irreversible electroporation of the truncus coeliacus, while the remaining two patients received portal vein and vena cava resection with anastomosis, respectively.

**Table 1:** Demographic, clinical and operative data of patients

Age (median and IQ range)	65 (60.25–70.25)
Sex (%)	
Male	8 (66.3%)
Female	4 (33.7%)
BMI (median and IQ range)	24.06 (20.90–26.15)
ASA score (%)	
1	0 (0%)
2	9 (75%)
3	3 (25%)
4	0 (0%)
Diabetes (%)	
Yes	3 (25%)
No	9 (75%)
Neoadjuvant chemotherapy (%)	
Yes	4 (33.7%)
No	8 (66.3%)
Pancreatic thickness on preoperative CT (median and IQ range)	20.15 (17.9–23.75)
Pancreatic consistency	
Soft	12 (100%)
Hard	0 (0%)
Vascular resection (%)	
Yes	3 (25%)
No	9 (75%)
Surgical approach (%)	
Open	8 (66.3%)
Laparoscopic	4 (33.7%)
Intraoperative crystalloid infusion (median and IQ range)	2000 (1000–3000)
Intraoperative blood losses (median and IQ range)	400 (200–700)
Operative time (median and IQ range)	144.50 (110.25–170.0)
Timing of drain removal (median and IQ range)	7 (7–12)
Length of hospital stay (median and IQ range)	13 (11–16.5)

*BMI*, body mass index; *ASA*, American Society of Anaesthesiology

Postoperative morbidity events are reported for each patient in Table 2. Overall, 2 (16.6%) patients experienced a grade 3 or higher surgical complication. We experienced only one case (8.3%) of POPF in our population: the fistula was classified as grade B and was managed with radiological drainage of the fluid collection. We did not experience any case of re-operation nor in-hospital mortality.

**Table 2:** Postoperative morbidity

Grade	Description	Management
4B	POPF B and anaphylactic reaction to tazobactam	Percutaneous drainage and ICU management
0		
0		
0		
0		
3A	Perihepatic abscess	Percutaneous drainage and antibiotic therapy
0		
2	Delayed gastric emptying	Nasogastric tube and TPN
0		
2	Delayed gastric emptying	Nasogastric tube and TPN
0		
0		

*TPN*, total parenteral nutrition

### Inferential analysis at univariate and bivariate levels

In our analysis, no preoperative variables showed statistically significant difference nor association with the development of pancreatic fistula or severe postoperative complications. Regarding intraoperative parameters, only median crystalloid infusion was significantly higher in patients who developed severe postoperative complications (4000 ml) than in those who did not (2000 ml) (*U* = 26.5; *z* = − 2.469; *p* = 0.009).

## Discussion

Despite a trend toward reduction in mortality in the latest years, a high rate of postoperative complications still causes a major burden for patients undergoing DP. Among these, POPF (and in particular grades B and C fistulas) still plays an important role in slowing down the postoperative recovery of these patients and possibly delaying the beginning of adjuvant therapies. However, consensus is still lacking regarding universally acknowledged risk factors for the development of POPF in this setting. In a recent review by Miyakasa et al. [16], risky features for POPF were divided into “patient-related” and “surgery-related” factors. The former included male gender, age, high BMI, soft pancreatic texture, chronic pancreatitis of the pancreatic remnant, low preoperative albumin levels, high ASA score, and smoking habits. *Surgery-related* risk factors involved prolonged duration of surgery, excessive blood losses, spleen preservation, non-ligation of the main pancreatic duct, transection at the tail, great volume of the remaining pancreas, extended lymphadenectomy and concomitant additional organ resection. Despite this effort to distinguish all the possible risk factors associated with development of POPF in DP, few studies report their fistula rates stratified according to the risk class of their subjects.

Following the insight about dangerous features associated with development of fistula, several strategies have been proposed in order to reduce the incidence of POPF after DP.

The first aspect to be investigated was the comparison between different techniques for the closure of pancreatic stump [1, 6, 7, 9, 16, 22, 28]. The majority of the literature reports no difference across various techniques: in particular, these studies claimed that the use of stapler did not increase the POPF rates [1, 7, 9, 28]. However, in favor of this latter approach, Ecker et al. [22] demonstrated a statistically significant lower rate of POPF after DP when transecting the pancreas with a stapler rather than with an energy device or by scalpel with subsequent handsewn closure of the pancreatic stump. Only the work by Kleef et al. [27] reported a significant higher rate of POPF after DP in the group of patient resected using a stapler; the reason may lay in small areas of teared pancreatic parenchyma that go unnoticed when using the stapler. Nevertheless, the same authors concluded that technical causes alone cannot be responsible for pancreatic fistula after DP. In the context of stapler use for transection and closure of the pancreatic stump, Hirashita et al. [33] demonstrated that a pre-firing compression of the transection plane for a total of 10 min was associated with a decreased rate of POPF after DP. These findings support the idea that the use of staplers in both open and close procedures is safe and can reduce the operative time by almost 1 hour [27].

Regarding the use of sealants, the review by Cheng et al. [34] and its most recent update [35] concluded that fibrin sealants may have little or no effect on postoperative pancreatic fistula in people undergoing DP.

Despite several studies reported controversial results about the administration of somatostatin analogs for prevention of POPF in pancreatic surgery [36, 37], some other scientific works support their use [38–40]. In particular, the randomized controlled trial by Allen et al. [41] demonstrated a significant decrease in POPF incidence also in the subgroup of patients undergoing DP. In their publication, Denbo et al. [42] concluded that high-risk and very high-risk patients are those profiting more from selective pasireotide administration.

The use of intraoperatively placed surgical drainages in order to reduce the incidence of POPF after DP has been thoroughly questioned. The rationale behind the use of these devices lays in their ability to remove pancreatic juices that leak from unsealed branches of the pancreatic duct, therefore avoiding their accumulation, which could lead to intra-abdominal abscess and bleeding [9]. Irrigating the pancreatic stump should dilute even minimal quantities of pancreatic juices, therefore reducing the risk of auto-activation of amylase and lipase, which would result in self-digestion of pancreatic gland. The benefits of this approach only in high-risk subjects have recently been demonstrated in animal models of mild and severe acute pancreatitis by Serra et al. [43]. In particular, the authors demonstrated that mortality was significantly reduced in severe acute pancreatitis animals with peritoneal lavage. At first sight, the technique described appears to be a mere mitigation strategy when a POPF is already in place, in order to decrease the impact of pancreatic juice on surrounding tissues. However, analyzing the problem in a different perspective, as authors we believe in the capacity of irrigation to prevent evolution of POPF from simple biochemical leaks to clinically-relevant pancreatic fistulas. In light of this, irrigation may actually result in a tangible lower incidence of grades B and C POPFs and their associated complications.

While some authors pointed out the lack of any significant difference in POPF rate between drain placement or not [44–46], other papers reported an increase in overall POPF when drainages were employed [47, 48]. However, the same authors recognized that the rates of CR-POPF did not differ between who received intraoperative drainage placement and who did not. These findings suggest that the use of surgical drainage allows the recognition of an increased total amount of low-grade fistulas, which would otherwise go unnoticed. In fact, according to the definition by the International Study Group on Pancreatic Fistula Definition [32], grade A fistulas have no clinical impact, and they require minimal change in management or deviation from the normal clinical pathway. In their study, Ecker et al. [48] demonstrated that the average complication burden for POPF was significantly lower when a drain was placed intraoperatively. Similar results were achieved by Machado et al. [45], in which an increased severity in POPF grade was observed in their control group. Seykora et al. [49] found a significant lower CR-POPF rate when drainages were removed early or in case they were not positioned at all. In a randomized trial published by Van Buren et al. [46], the use of drainage was not associated with any difference in CR-POPF rates or mortality; nevertheless, the no-drain group experienced a higher intra-abdominal fluid collection rates.

When compared with the literature, our major complications rate of 16.6% and the POPF rate of 8.3% seem very promising results, especially considering that they refer to a population at very high risk to develop postoperative fistulas and complications. The few studies reporting stratified outcome assessed a CR-POPF rate from 11.2 to 59% [5, 11, 14, 42] and a major complication rate between 16.6 and 24.7% [11, 14] in the high-risk population. Optimism also comes from the null mortality rate in our series of patients, which becomes more encouraging especially when compared with the 44% 30-day mortality for patient with more than 4 points according to the score proposed by Kelly et al. [4]. A certain overlap exists also in terms of mean length of hospitalization between our patients and the one reported by Vanbrugghe et al. [5] (14.6 vs. 18.4 days).

To our knowledge, the paper published by Bu et al. [50] is the first to report the use of irrigation around the pancreatic stump after DP. In this study, the irrigation of the pancreatic stump did not reduce the rate of POPF; however, patients with CR-POPF and receiving irrigation manifested better clinical conditions and required less specific treatment than those in the control group. However, the Chinese group did not include any distinction between patients at high or low risk to develop POPF. Together with our findings, we are confident about the usefulness of this particular drainage approach only in high-risk patients.

Nevertheless, our paper has several limitations. The retrospective nature of this study, but most importantly the very limited number of high-risk subjects undergoing DP, did not allow us to reach consistent conclusions under the statistical point of view. Despite the wide number of variables tested against two different outcomes, we were able to only identify the association between intraoperative crystalloid infusion and development of postoperative complications. Therefore, we aim to improve the number of subjects included in the analysis along with time. Another drawback is the lack of a control group. Unfortunately, a few data were available for patients operated before the introduction of Salem sump drainages and when present, they were of poor quality. Therefore, it was impossible to extract data regarding a historical high-risk control group in order to attempt a comparison about the effectiveness of the newly introduced drain management for prevention of POPF.

## Conclusions

Despite those limitations and the modesty of our results, we strongly believe that the postoperative course of high-risk patients undergoing DP can be improved by this new drainage management. To further develop insight about the potentiality of irrigation with passive drainage, we firstly need consensus and validated models to classify patients at high risk in an objective and standardized way. Once this will be achieved, we will propose our hypothesis-generating study as the starting point for a possible multicenter randomized trial.

## Data Availability

The dataset used during the current study are available from the corresponding author on reasonable request.
